# Total joint arthroplasty for thumb carpometacarpal joint osteoarthritis: a systematic review and meta-analysis of randomized controlled trials

**DOI:** 10.2340/17453674.2024.40816

**Published:** 2024-06-18

**Authors:** Rasmus LIUKKONEN, Venla-Linnea KARJALAINEN, Reetta KVIST, Matias VAAJALA, Ville PONKILAINEN, Teemu KARJALAINEN

**Affiliations:** 1 Faculty of Medicine and Health Technology, Tampere University, Tampere; 2 Central Finland Central Hospital Nova, Jyväskylä; 3 Department of Hand and Microsurgery, Tampere University Hospital Tampere, Finland

## Abstract

**Background and purpose:**

Thumb carpometacarpal (CMC) joint osteoarthritis (OA) is increasingly treated with total joint arthroplasty (TJA). We aimed to perform a systematic review and meta-analysis of the benefits and harms of the TJA for thumb CMC OA compared with other treatment strategies.

**Patients and methods:**

We performed a systematic search on MEDLINE and CENTRAL databases on August 2, 2023. We included randomized controlled trials investigating the effect of TJA in people with thumb CMC joint OA regardless of the stage or etiology of the disease or comparator. The outcomes were pooled with a random effect meta-analysis.

**Results:**

We identified 4 studies randomizing 420 participants to TJA or trapeziectomy. At 3 months, TJA’s benefits for pain may exceed the clinically important difference. However, after 1-year follow-up TJA does not improve pain compared with trapeziectomy (mean difference 0.53 points on a 0 to 10 scale; 95% confidence interval [CI] 0.26–0.81). Furthermore, it provides a transient benefit in hand function at 3 months (measured with Disabilities of Arm, Shoulder, and Hand questionnaire, scale 0–100, lower is better) compared with trapeziectomy with or without ligament reconstruction tendon interposition. The benefit in function diminished to a clinically unimportant level at 1-year follow-up (4.4 points better; CI 0.42–8.4).

**Conclusion:**

Transient benefit in hand function for TJA implies that it could be a preferable option over trapeziectomy for people who consider fast postoperative recovery important. However, current evidence fails to inform us if TJA carries long-term higher risks of revisions compared with trapeziectomy.

Osteoarthritis (OA) of the carpometacarpal (CMC) joint of the thumb is a common condition affecting the hands of middle-aged and elderly people. The prevalence of this condition is around 15% within the female population, and around 7% within the male population [1].

For people with persistent symptoms after nonoperative treatment, several surgical strategies have been proposed. Traditionally, the most common procedure to treat CMC OA is a trapeziectomy with or without ligament reconstruction tendon interposition (LRTI) arthroplasty [2-5]. Arthrodesis has also been used, but it seems to carry a higher risk of adverse effects without benefits [6]. Various prosthetic implants have also been introduced, including total joint arthroplasties (TJAs) of several designs [5,7]. Purported benefits of TJA include avoidance of metacarpal collapse typical of trapeziectomy [8], but, on the other hand, the implant increases primary costs and involves a risk of loosening or dislocation [9,10]. The risk of reoperation might be higher for TJA, as in a 10-year follow-up, where the implant survival was estimated to be around 90% [11].

To date, the decision to perform trapeziectomy or TJA has largely depended on surgeons’ preferences [5]. Recent randomized controlled trials (RCT) have compared trapeziectomy and TJA in the treatment of CMC OA, but none of them has been sufficiently large to conclude on the benefit of 1 procedure over the other [12-15]. Thus, we aimed to perform a systematic review and meta-analysis of the benefits and harms of the TJA for thumb CMC OA compared with other treatment strategies.

## Methods

We adhered to the PRISMA guidelines throughout the study [16].

### Identification of studies

We included all randomized, quasi-randomized controlled trials, or studies that compared thumb CMC TJA with any surgical, non-surgical, or placebo treatment in participants with CMC joint OA, regardless of the stage or etiology of the disease. Searches were conducted in MEDLINE and CENTRAL databases on August 2, 2023. The protocol defined comparative observational studies as potentially eligible for inclusion. However, because several RCTs were identified, and non-randomized trials are at high risk of bias, we decided to include only RCTs in the analyses.

### Study selection

2 authors independently screened the titles and abstracts and reviewed the full texts of potentially eligible studies. Disagreements were resolved through discussion.

### Outcome measures

The main outcome is pain. In cases where multiple pain outcomes were reported in the same trial, we prioritized the Visual Analogue Scale (VAS), which yields a score of 0 to 10, with a higher score indicating more pain. If various pain outcomes were reported, we prioritized pain with activity or overall pain instead of rest pain.

Secondary outcomes were hand or thumb function, as measured by a validated patient reported outcome measure (PROM) or any other self-reported measure; grip strength; pinch strength; overall improvement or satisfaction; Health-Related Quality of Life (HRQoL); adverse events; reoperation; and return to work. The Disability of the Arm, Shoulder, and Hand Questionnaire (DASH) was prioritized as the PROM, as it was the most used measure.

### Data collection and handling

The predefined time points were 3 months, 1 year, and 2 to 3 years. Since none of the studies followed participants for longer than 2 years, we collected and analyzed 2-year data.

All data was extracted to a pretested pro forma. This included the name of the first author, year of publication, number of participants, intervention and control treatments, and outcomes at each time point. Adverse events, reoperation, and satisfaction were collected at the latest available follow-up. We preferred data from the intention-to-treat analysis, but if not reported, we used per-protocol or as-treated data.

### Risk of bias and certainty of evidence

2 authors independently assessed the risk of bias based on the Cochrane risk of bias tool 1.0 [17]. Disagreements were resolved through discussion and, if necessary, by consulting a third author. We evaluated the certainty of evidence using the GRADEpro tool [18]. We downgraded the certainty reflecting our confidence in the treatment effect based on the following factors: (i) risk of bias (downgraded if included studies were at high or unclear risk of bias), (ii) inconsistency (downgraded if the studies in the meta-analyses were inconsistent without apparent explanation), (iii) imprecision of the estimates (considered precise when the 95% confidence intervals (CIs) excluded or supported clinically significant benefits), (iv) indirectness of the evidence (present when surrogate outcomes were used). We did not assess publication bias due to the low number of studies available for each meta-analysis.

### Statistics

For continuous outcomes, we extracted the mean and standard deviation (SD) values. If the mean was not available, we approximated it with the median. When SD was unavailable, we calculated it based on the interquartile range (IQR). For binary outcomes, we extracted events and the total number of participants at each time point. When total numbers were not available at each time point, we used the number of participants randomized to each group.

All meta-analyses were stratified by intervention. We used inverse variance weighting with a random effects model in all meta-analyses. For continuous outcomes, the treatment effect was expressed as mean difference (MD) with CIs. For binary outcomes, we expressed the treatment effect as relative risk (RR) with CIs. When different measures were used for one outcome domain, we used standardized mean difference (SMD) for pooling and then back-translated the SMD to the original scale using typical SD. If studies reported both binary and continuous measures for an outcome, we transformed the odds ratio [19] to SMD and used SMD as the summary measure.

We estimated whether the benefit was patient-important based on the CI of the estimate and the minimal clinically important difference (MCID) value of the corresponding outcome measure. The heterogeneity of the meta-analysis was determined through visual inspection of the forest plots and I^2^ statistics. We used the packages meta and metafor in R (4.3.0) with the software RStudio (2023.03.0+386) for the meta-analyses [20-23].

### Ethics, registration, funding, and disclosures

Due to the study setting, no institutional review board approval was required. This systematic review was prospectively registered in the PROSPERO database (CRD42023451019). No funding was received for this study. The authors have nothing to disclose. Complete disclosure of interest forms according to ICMJE are available on the article page, doi: 10.2340/17453674.2024.40816

## Results

### Literature search and study selection

**Figure 1:**
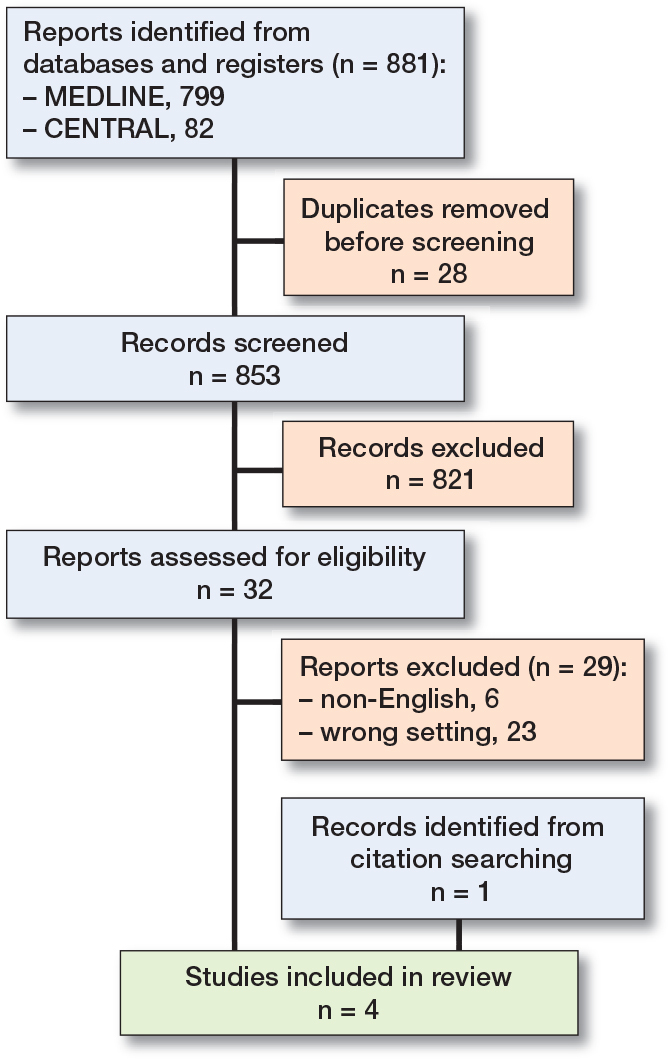
PRISMA flowchart of the study selection process. PRISMA = Preferred Reporting Items for Systematic Reviews and Meta-Analyses.

The search yielded 881 records, of which 849 were excluded in the initial screening process. In addition, 1 potential study was identified by citation searching [15]. After reading 33 full texts, we included 4 eligible studies (Figure 1).

The studies were published in 2019 and 2023 and are summarized in Table 1. 3 studies [12,13,15] compared TJA with trapeziectomy and LRTI, and 1 study [14] compared TJA with trapeziectomy without LRTI. We did not identify any studies that compared TJA with a nonoperative treatment or placebo.

**Table 1:** Characteristics of included studies

First author, year	Country	Participants	Mean age	Women (%)	Control intervention	Outcomes collected	Follow-up months
de Jong, 2023	Netherlands	62	60	100	Trapeziectomy	Pain (MHOQ subcale), DASH score, grip strength, pinch strength, satisfaction, reoperation, adverse events	12
Guzzini, 2023	Italy	150	67	75	Trapeziectomy + IA	Pain (VAS), DASH score, grip strength, pinch strength, satisfaction, reoperation, adverse events	24
Klim, 2023	Austria	168	58	81	Trapeziectomy + IA	Pain (VAS), DASH score, HADS score, satisfaction, reoperation	12
Thorkildsen, 2019	Norway	40	63	70	Trapeziectomy + IA	QuickDASH score, grip strength, pinch strength, satisfaction, reoperation, adverse events	24

MHOQ subscale = Michigan Hand Outcomes Questionnaire, pain subscale.

DASH = Disability of the Arm, Shoulder, and Hand Questionnaire, scale 0–100, lower better.

IA = interposition arthroplasty.

Pain VAS = visual analog scale (0–10, lower is better).

HADS = Hospital Anxiety and Depression Scale 0–42, lower is better.

QuickDASH = short version of DASH, scale 0–100, lower is better.

### Pain

At 3 months and 1 year, 2 studies [13,15] measured pain using VAS, and 1 [14] used the Michigan Hand Outcomes Questionnaire. We considered 1.5 points to represent MCID in VAS [24].

**Figure 2:**
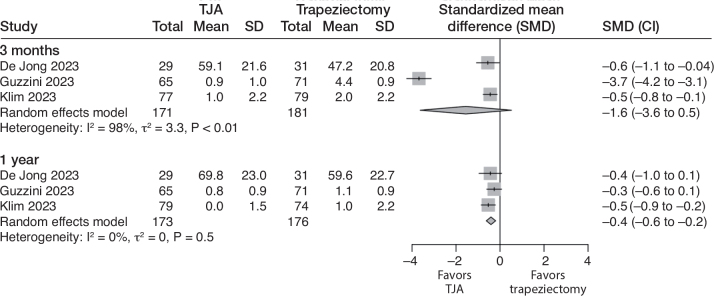
Forest plot for pain. Results are reported separately after 3-month and 1-year follow-up. TJA = total joint arthroplasty, SD = standard deviation, CI = confidence interval.

At 3 months, TJA seemed to provide a clinically relevant benefit in terms of pain, but the evidence is very uncertain (downgraded due to risk of bias, inconsistency, and imprecision). The SMD was –1.6 (CI –3.6 to 0.5; 3 studies; 352 participants; I^2^ = 98%). This translates to 2.0 (CI –0.7 to 4.7) VAS points better for TJA compared with trapeziectomy (Table 2, Figure 2).

**Table 2:** GRADE summary findings for comparison of total joint arthroplasty with trapeziectomy

Outcome, time frame	Measurement instruments and relative effects ^a^	Effect estimates		Certainty of evidence ^b^ (reasons)	Plain text summary
		Trapeziectomy (absolute score or risk)	TJA (mean difference or RR)		
Pain, 3 months	VAS		SMD –1.6 (CI –3.6 to 0.5)	Very low (inconsistency, imprecision)	Uncertainty about the effect
3 studies	MCID: 1.5 points		Translates to		
352 patients			2.0 (CI –0.7 to 4.7) VAS points better		
Pain, 1 year	VAS		SMD –0.4 (CI –0.6 to –0.2)	Moderate	TJA does not provide important pain reduction at 1 year
3 studies	MCID: 1.5 points		Translates to		
349 patients			0.5 (CI 0.3 to 0.8) VAS points better		
Function, 3 months	DASH	39	18 (CI 15 to 21) points better	Moderate	TJA improves function at 3 months
4 studies	MCID: 11 points				
385 patients					
Function, 1 year	DASH	16	4.4 (CI 0.4 to 8.4) points better	Moderate	TJA probably improves function at 1 year
4 studies	MCID: 11 points				
380 patients					
Grip strength, 3 months	–	16 kg	3.7 (CI 1.7 to 5.8) kg better	Low (imprecision)	TJA may improve grip strength at 3 months
3 studies	MCID: 5–6.5 kg				
227 patients					
Grip strength, 1 year	–	23 kg	2.1 (CI –4.0 to 8.1) kg better	Very low, (inconsistency imprecision)	Uncertainty about the effect at 1 year
3 studies	MCID: 5–6.5 kg				
226 patients					
Pinch strength, 3 months	–	3.8 kg	1.5 (CI 0.7 to 2.4) kg better	Low (imprecision)	TJA may improve pinch strength at 3 months
3 studies	MCID: 0.35 kg				
228 patients					
Pinch strength, 1 year	–	4.8 kg	0.9 (CI 0.2 to 1.6) kg better	Very low (inconsistency, imprecision)	TJA may improve pinch strength at 1 year
3 studies	MCID: 0.35 kg				
226 patients					
Global satisfaction			SMD –0.42 (CI –0.89 to 0.06)	Very low (inconsistency, imprecision)	Uncertainty about the effect
4 studies					
395 patients					
Reoperation		6/208	RR 2.0 (CI 0.11 to 36)	Very low (inconsistency, very serious imprecision)	Uncertainty about the effect
4 studies					
406 patients					
(de Jong et al. excluded)		0/177	RR 9.1 (CI 1.2 to 70)	Moderate	Probably higher risk of reoperation after TJA
3 studies					
344 patients					
Adverse events		36/122	RR 0.9 (CI 0.6 to 1.4)	Low (imprecision)	Uncertainty whether TJA causes fewer adverse events
3 studies					
238 patients					
Return to work		7 weeks	6 weeks		Uncertainty about the effect
1 study					
Quality of life, 3 months	No data				
Quality of life, 1 year	No data				

a VAS = visual analog pain scale (0–10, lower is better); MCID = minimal clinically important difference; DASH = Disability of the Arm, Shoulder, and Hand Questionnaire, scale 0–100, lower is better.

b Evidence was downgraded 1 step for all estimates because all studies were in high risk of bias.

At 1 year, the benefits of TJA are not clinically important, as indicated by moderate certainty evidence (downgraded due to risk of bias). The SMD was –0.4 (CI –0.2 to –0.6; 3 studies; 349 participants; I^2^ = 0%), translating to a VAS of 0.5 (CI 0.3–0.8) point better with TJA.

At 2 years, one study [15] found no clinically important benefit for TJA. The mean VAS score was 1.1 in the trapeziectomy group and 0.4 (CI 0.1–0.7) points better with TJA (1 study; 136 participants). The certainty of the evidence was downgraded to low due to the risk of bias and imprecision (only 1 study with a low number of participants).

### PROMs

**Figure 3:**
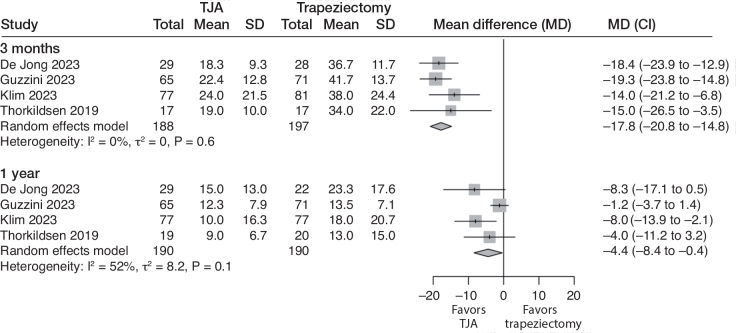
Forest plot for DASH score. Results are reported separately after 3-month and 1-year follow-up. DASH = Disabilities of the Arm, Shoulder, and Hand. See legend to Figure 2 for further abbreviations.

All 4 studies measured PROMs using DASH or QuickDASH (0–100, lower is better; MCID of 10.8 points in DASH; MCID of 11 in QuickDASH [25]) at 3 months and 1 year. At 3 months, moderate certainty evidence (downgraded for risk of bias) indicates that the TJA improves hand function. The mean DASH score was 39 points with trapeziectomy and 18 (CI 15–21) points better with TJA (4 studies; 385 participants; I^2^ = 0%) (Figure 3).

At 1 year, moderate certainty evidence (downgraded for risk of bias) indicates that there is no clinically relevant benefit for TJA. The mean DASH score was 16 with trapeziectomy and 4.4 (CI 0.4–8.4) points better with TJA (4 studies; 380 participants; I^2^ = 52%).

At 2 years, data from 2 studies [12,15] indicated that TJA did not improve hand function compared with trapeziectomy. The mean DASH score was 16 with trapeziectomy and 3.8 (CI–3.4 to 11) points better with TJA (2 studies; 172 participants; I^2^ = 59%). The certainty of the evidence was low due to the risk of bias and imprecision.

### Grip strength

3 studies [12,14,15] measured grip strength (kg) at 3 months and 1 year. At 3 months, low certainty evidence (downgraded for risk of bias and imprecision) indicates no clinically relevant difference between TJA and trapeziectomy (MCID of 5–6.5 kg in grip strength [26]). Mean grip strength was 16 kg with trapeziectomy and 3.7 (CI 1.7–5.8) kg better with TJA (3 studies; 227 participants; I^2^ = 0%).

At 1 year, we are uncertain of the effect (very low certainty). The mean grip strength was 23 kg with trapeziectomy and 2.1 (CI –4.0 to 8.1) kg better with TJA (3 studies; 226 participants; I^2^ = 83%). The certainty of the evidence was downgraded due to the risk of bias, inconsistency, and imprecision.

At 2 years, low certainty evidence (downgraded due to risk of bias and imprecision) indicates that TJA seems to improve grip strength compared with trapeziectomy with or without LRTI. The mean grip strength was 25 kg with trapeziectomy and 6.3 (CI 3.7–8.9) kg better with TJA (2 studies; 172 participants; I^2^ = 0%).

### Pinch strength

3 studies [12,14,15] measured pinch strength (kg) at 3 months and 1 year. Low certainty evidence (downgraded due to risk of bias and inconsistency) suggests that the benefits of TJA compared with trapeziectomy are greater than the MCID of 0.35 kg in pinch strength [27]. Mean pinch strength was 3.8 kg with trapeziectomy and 1.5 (CI 0.7–2.4) kg better with TJA (3 studies; 228 participants; I^2^ = 82%).

At 1 year, mean pinch strength was 4.8 kg with trapeziectomy and 0.9 (CI 0.2–1.6) kg better with TJA (3 studies; 226 participants; I^2^ = 73%). The certainty of the evidence was downgraded to very low due to the risk of bias, imprecision, and inconsistency.

At 2 years, 2 studies [12,15] measured pinch strength. Moderate certainty evidence (downgraded due to risk of bias) indicates that the TJA provides a clinically significant benefit in pinch strength over trapeziectomy. Mean pinch strength was 5.2 kg in the trapeziectomy group and 1.4 (1.2–1.7) kg better with TJA (2 studies; 172 participants; I^2^ = 0%).

### Satisfaction

2 studies [14,15] reported satisfaction using a numeric rating scale that yields scores from 0 to 10. Thorkildsen and Røkkum [12] reported satisfaction as a binary outcome. Klim et al. [13] reported satisfaction as a scale with 4 steps from “very satisfied” to “dissatisfied.” To allow pooling, we collected it as a binary outcome with “very satisfied” or “satisfied” indicating satisfaction and transformed the effect to SMD. The SMD was –0.42 (CI –0.9 to 0.1; 4 studies; 395 participants; I^2^ = 63%), favoring TJA. The certainty of the evidence was downgraded to very low due to the risk of bias, inconsistency, and imprecision.

### Reoperation

**Figure 4:**
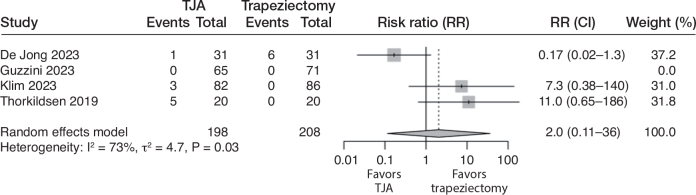
Forest plot for risk of reoperation. See legend to Figure 2 for abbreviations.

All 4 studies reported reoperation rates. In the TJA group, 9/198 (5%) were reoperated, while 6/208 (3%) were reoperated in the trapeziectomy group. This corresponds to a risk ratio of 2.0 (CI 0.11–36; 4 studies; 406 participants; I^2^ = 73%) in favor of trapeziectomy. The certainty of evidence was downgraded to very low due to the risk of bias, inconsistency, and very serious imprecision (the CIs were consistent with large benefits in either direction; Figure 4).

Due to great inconsistency and imprecision in reoperation, we additionally report the pooled results excluding the study by de Jong et al. In the remaining studies, 8/167 (5%) were reoperated in the TJA group and 0/177 (0%) in the trapeziectomy group. The pooled risk ratio was 9.1 (CI 1.2–70; 3 studies; 344 participants; I^2^ = 0%) in favor of trapeziectomy. In this analysis, the certainty of the evidence was downgraded to moderate due to the risk of bias.

### Adverse events

**Figure 5:**
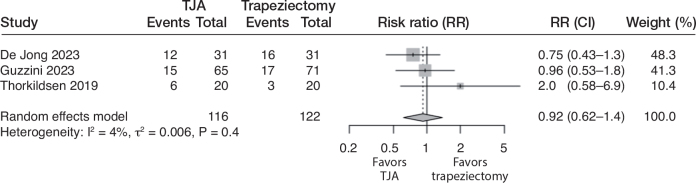
Forest plot for adverse events. See legend to Figure 2 for abbreviations.

3 studies [12,14,15] reported adverse events. There were 33/116 (28%) adverse events in the TJA group and 36/122 (30%) in the trapeziectomy group. There was no difference in pooled risk ratio, which was 0.9 (CI 0.6–1.4; 3 studies; 238 participants; I^2^ = 4%). The certainty of the evidence was downgraded to low due to the risk of bias and imprecision (Figure 5).

### Return to work

Only 1 study [14] reported a return to work so a meta-analysis was not performed. The study found no statistically significant difference between the treatments in return to work. The median time off from work in the TJA group was 6 weeks (IQR 1–10) compared with 7 (IQR 4.5–9) in the trapeziectomy group .

### Health-related quality of life (HRQoL)

No studies measured generic HRQoL. 1 study [13] reported the Hospital Anxiety and Depression Scale (HADS) questionnaire as quality of life. At 3 months, the median HADS score (0–42, lower is better) was 8 (IQR 4–14) with trapeziectomy and 5 (IQR 1–12) with TJA (P = 0.2). At 1 year the score was 8 (IQR 2–16) in the trapeziectomy group and 5 (IQR 1–12) in the TJA group (P = 0.2).

## Discussion

We aimed to perform a systematic review and meta-analysis to estimate the benefits and harms of TJA for thumb CMC OA compared with other treatment strategies.

We found that pain relief is comparable between TJA and trapeziectomy at 1 year. TJA, however, seems to provide clinically relevant benefits in hand function compared with trapeziectomy in the short term. This benefit diminishes by 1-year follow-up to a clinically negligible level, but grip and pinch strength may still be higher with TJA compared with trapeziectomy, suggesting possible long-term benefits over trapeziectomy. We found no evidence of harms for TJA compared with trapeziectomy but our confidence at both risk for reoperation and adverse effect is constrained by imprecise estimates and disparities among study findings.

Concerning pain, our conclusions diverge from prior reviews, likely due to an exclusive focus on RCTs and our utilization of a systematic (GRADE) approach to evaluate the certainty of evidence [28,29]. Furthermore, the analyses in the previous reviews were not stratified by the duration of the follow-up, making direct comparison unfeasible. Regarding function, our findings are in line with the previous reviews [28,29]. However, the previous reviews included mostly observational studies, rendering the conclusions less reliable. As 3 of the 4 RCTs in this review have been published recently, they were not included in the previous reviews.

TJA has been associated with a higher risk of complication and reoperations, limiting its popularity [28,29]. Froschauer et al. reported only a 60% 5-year survival rate [30]. Recent observational studies, however, suggest a 5-year survival rate up to 96% [31]. We considered the evidence for the reoperation rate to be too uncertain to draw firm conclusions because the included studies were at risk of bias, reported inconsistent findings, and the risk estimates were imprecise. By removing one outlier study, the reoperation rate was lower with trapeziectomy than with TJA. Inconsistency may relate to a threshold to intervene and is likely not a reliable outcome in unblinded studies. After a failed TJA, trapeziectomy is still a viable option, but after a failed trapeziectomy, reoperations are considered unreliable at improving symptoms [32].

Because the primary costs of TJA are higher than trapeziectomy, the benefits and harms of these procedures should be evaluated carefully before making clinical guidelines. As many of the estimates were of low or very low certainty, large, blinded trials are needed. The better functional outcomes after TJA, and the possibility of lower pain, might imply shorter intervals between operation and returning to work, although this review could not confirm this due to lack of data. Therefore, the working-age population may be a population of particular interest in future trials.

### Limitations

We identified no studies comparing TJA with nonoperative treatment, no treatment, or placebo treatment. Therefore we were not able to answer the question as to whether TJA is better than nonoperative treatments. The evidence comes from trials that were at high risk of bias and sometimes inconsistent, limiting our certainty to the estimates. This inconsistency may relate to different prosthesis designs, but current data is too limited for stratified analyses. Furthermore, many of the estimates were imprecise, indicating a lack of information for some of the outcomes such as risk of adverse effects and reoperation. We searched the databases systematically but were unable to assess the publication as since too few studies were included. Studies with unfavorable results may have been left unpublished, biasing the pooled estimates in this study. One main limitation of the present study is that there are no high-quality studies on the long-term outcomes between TJA and trapeziectomy.

### Conclusion

Transient benefit in hand function for TJA implies that it could be a preferable option over trapeziectomy for people who consider fast postoperative recovery important. However, current evidence fails to inform us whether TJA carries long-term higher risks of revisions compared with trapeziectomy, which might impact decision-making. Further rigorous blinded trials with long-term follow-up are warranted.

## Notes

All authors: interpretation of results, manuscript editing, and approval of the final version. RL: data collection, statistical analyses, writing of the first version of the manuscript. VLK: data collection, statistical analyses. RK and MV: data collection. VP and TK: study planning, project supervision.

Handling co-editors: Li Felländer-Tsai and Robin Christensen

*Acta* thanks Maria Wilcke and Malin Zimmerman for help with peer review of this manuscript.
